# Antibacterial Activity of Rosmarinic Acid Against *Streptococcus agalactiae* and Its Anti-Inflammatory Effects in Mouse Mastitis

**DOI:** 10.3390/ani16182917

**Published:** 2026-09-16

**Authors:** Jishang Gong, Xuewen Chai, Yanbei Yang, Xin Yang, Haoxiang Xu, Jiguo Xu, Xinwei Xiong, Yuanfei Li

**Affiliations:** Institute of Biological Technology, Nanchang Normal University, Nanchang 330032, China; gongjishang@126.com (J.G.); chaixuewen03@ncnu.edu.cn (X.C.); yangyanbei1986@163.com (Y.Y.); xinyang2498@foxmail.com (X.Y.); xhx1002006@126.com (H.X.); xujiguo@ncnu.edu.cn (J.X.); xinweixiong@hotmail.com (X.X.)

**Keywords:** RA, *Streptococcus agalactiae*, antibacterial, anti-inflammatory

## Abstract

Mastitis severely hinders dairy farming, with *Streptococcus agalactiae* (*S. agalactiae*) serving as a major causative pathogen. Prolonged antibiotic administration promotes the emergence of multidrug-resistant strains, thereby compromising mastitis control. Consequently, novel natural antibacterial agents are urgently needed. In this study, bacteriostatic assays identified the MIC of rosmarinic acid (RA) against *S. agalactiae* as 8 mg/mL. At the MIC, RA reduced the adhesion capacity of *S. agalactiae* by 16.6% and inhibited biofilm formation by 25.09%. RA also disrupted bacterial membrane architecture and markedly downregulated the expression of virulence factors. A mouse model of mastitis treated with varying doses of RA (25, 50, and 100 mg/kg) indicated that RA attenuated pathological damage to the mammary duct architecture and ameliorated inflammatory responses in *S. agalactiae*-infected mice. ELISA revealed that RA significantly reduced pro-inflammatory mediators, including IL-1α, IL-1β, IL-6, and TNF-α. RT-qPCR results indicated that mRNA expression levels of TLR2, NF-κB, AKT, and PI3K were substantially reduced following RA treatment, whereas apoptosis-related genes p53, RUNX1, and Bim exhibited distinct expression patterns. Western blotting analysis further indicated that RA modulated the phosphorylation of Bim, RUNX1, and p53 in mastitis tissues, suppressed the TLR2/NF-κB inflammatory signaling pathway, and regulated the PI3K/AKT cascade. By targeting multiple pathways, RA suppressed *S. agalactiae* proliferation and attenuated mammary gland inflammation, highlighting its potential as a green therapeutic agent for sustainable animal husbandry.

## Simple Summary

Mastitis is a prevalent and severe disease that hinders the development of the dairy industry. Improper use of antibiotics in dairy farms frequently promotes pathogen resistance, posing significant risks to animal production and food safety. Consequently, the development of natural, environmentally friendly antibacterial and anti-inflammatory alternatives is of high practical importance. Rosmarinic acid (RA), a natural plant extract, exhibits potential antibacterial and anti-inflammatory activities. This study evaluated the antibacterial and anti-inflammatory effects of RA against *Streptococcus agalactiae* (*S. agalactiae*) using bacterial assays and a mouse model of mastitis. Bacterial assays indicated that RA inhibited virulence factor expression and biofilm formation in *S. agalactiae*. Mouse mastitis experiments revealed that RA alleviated mammary inflammation by modulating the TLR2/NF-κB and PI3K/AKT signaling pathways. In conclusion, RA suppresses the growth of *S. agalactiae* and attenuates *S. agalactiae*-induced mouse mastitis. These findings provide a theoretical foundation for the development of novel green therapeutic strategies against mastitis and offer insights for sustainable livestock production.

## 1. Introduction

Mastitis is a prevalent disease in dairy cows that serves as a primary cause of reduced milk yield [1]. The condition results in substantial economic losses worldwide, with annual management costs in Europe and North America reaching billions of US dollars [1,2]. Mastitis manifests in either clinical or subclinical forms, with clinical cases frequently developing from unmanaged subclinical infections. Subclinical mastitis is primarily characterized by an elevated somatic cell count (SCC) in milk and often evades routine clinical detection, requiring specialized diagnostic techniques [3,4]. Infectious pathogens driving bovine mastitis include *Staphylococcus aureus* (*S. aureus*), *Streptococcus agalactiae* (*S. agalactiae*), and *Mycoplasma* spp. [5]. These microorganisms are commonly transmitted among healthy cows during milking via contaminated equipment, towels, or handlers. *S. agalactiae* represents a major pathogen in subclinical mastitis, contributing significantly to industry-wide economic losses [6]. The virulence factors of *S. agalactiae* facilitate adhesion to and invasion of host epithelial cells, promoting biofilm formation that impairs host immune functions and accelerates pathology [7]. *S. agalactiae* encodes diverse proteins essential for colonization, invasion, biofilm formation, and immune evasion. Key structural determinants, including fibronectin-binding proteins, laminin-binding proteins, and pili, mediate critical interactions with host cell receptors and the extracellular matrix during mammary tissue invasion [8,9,10,11]. Beyond veterinary health, *S. agalactiae* poses a significant public health threat by causing severe neonatal infections, septicemia, and high-mortality meningitis in humans [12]. Gong et al. indicated that *S. agalactiae* virulence factors modulate RUNX1 and p53 signaling in host mammary epithelial cells to exacerbate inflammatory responses [13]. Therefore, controlling *S. agalactiae*-induced mastitis is essential for protecting livestock economics and safeguarding public health.

Antibiotics remain the primary intervention for bacterial mastitis in dairy cattle. Antibiotics remain the primary intervention for bacterial mastitis in dairy cattle. However, improper and excessive antibiotic administration has accelerated antimicrobial resistance (AMR), led to rumen dysbiosis, caused antibiotic tissue residues, and lowered milk quality. The escalation of AMR has spurred interest in natural bioactive compounds as alternative therapeutic options [14]. Botanical extracts from rosemary, ginkgo, chamomile, and garlic-containing compounds such as rosmarinic acid (RA), quercetin, and thymol enhance animal immune responses and reduce disease susceptibility [15]. RA is a natural phenolic ester of caffeic acid and 3,4-dihydroxyphenyllactic acid (DHPL) isolated from Rosmarinus officinalis and various other plant species [16]. It exhibits broad-spectrum biological activities, including anti-inflammatory, antioxidant, antimicrobial, and antiviral properties [17]. Previous studies show that RA inhibits both Gram-positive and Gram-negative bacteria at specific minimum inhibitory concentrations (MICs) [16,18]. Specifically, RA restricts *S. aureus* growth by inhibiting biofilm formation, disrupting Na^+^/K^+^-ATPase activity, increasing cell membrane permeability, and suppressing DNA polymerase activity [18,19,20,21]. Furthermore, Jiang et al. demonstrated that RA attenuates lipopolysaccharide (LPS)-induced mastitis by inhibiting the TLR4/MyD88/NF-κB signaling pathway [22].

Despite these findings, existing research on RA in mastitis models has largely focused on LPS-induced damage or *S. aureus* infection. Information regarding the direct action of RA against *S. agalactiae* remains limited. Specifically, whether RA influences *S. agalactiae* virulence gene expression, cell adhesion, biofilm formation, or host–pathogen signaling networks is poorly understood. Therefore, this study was designed to comprehensively evaluate the antibacterial and anti-inflammatory properties of RA against *S. agalactiae* using both bacterial assays and a mouse mastitis model. Our findings offer insights into the mechanistic potential of RA as a green therapeutic alternative for mastitis prevention and management.

## 2. Materials and Methods

### 2.1. Study Timeline

The study was conducted in two sequential stages. In Stage 1, the antibacterial activity of RA against *S. agalactiae* was evaluated in vitro. First, the minimum inhibitory concentration (MIC) of RA against *S. agalactiae* was determined in September 2024. Subsequently, the effects of different concentrations of RA on bacterial adhesion to MAC-T mammary epithelial cells and biofilm formation were evaluated in September 2024. *S. agalactiae* treated with RA at the MIC was harvested for TEM observation (TECNAI G 20 TWIN (FEI Company, Hillsboro, OR, USA)) of its biofilm in October 2024. *S. agalactiae* exposed to RA at the MIC was then harvested for RT-qPCR analysis of virulence-associated genes, including genes related to adhesion, invasion, immune evasion, capsular polysaccharide synthesis, and pilus formation in October 2024.

In Stage 2, the anti-inflammatory and tissue-protective effects of RA were evaluated in a postpartum/lactating mouse model of *S. agalactiae*-induced mastitis. Mastitis was established by intramammary infection with *S. agalactiae* and verified by qRT-PCR analysis of inflammatory markers in March 2025. The mice were then allocated to a control group, an infected model group, and three RA treatment groups receiving different doses of RA. Following treatment, milk, peripheral blood, and mammary gland tissues were collected for subsequent analyses in April 2025. Milk samples were used for somatic cell counting (in April 2025), peripheral blood was analyzed for immune-cell profiles (in April 2025), and mammary gland tissues were subjected to histopathological examination using H&E staining in May 2025. Inflammatory cytokines were quantified by ELISA, while changes in gene and protein expression were assessed by qRT-PCR and Western blotting (May 2025), respectively.

Together, the two-stage experimental design enabled the antibacterial effects of RA on *S. agalactiae* and its anti-inflammatory and tissue-protective effects in infected mammary tissues to be evaluated in a complementary manner.

### 2.2. Animals and Cell Lines

Thirty female Kunming mice (12 weeks old; 30–35 g) were provided by Nanjing Kangxuhe Biotechnology Co., Ltd. (Nanjing, China). All procedures were approved by the Animal Ethics Committee of Nanchang Normal University (No. 2025002), and adhered strictly to “Regulations for the Administration of Laboratory Animals in China”, as well as the guidelines sanctioned by the Animal Management Committee of Nanchang Normal University. Throughout the experiment, the animals were housed in plastic cages at a temperature of 20–26 °C and a humidity of 50–60% under a 12 h/12 h light/dark cycle, with unrestricted access to food and water. MAC-T cells were provided by the College of Life Science and Technology, Gansu Agricultural University (Lanzhou, China). *S. agalactiae* strain ATCC 27956 was preserved by our laboratory.

### 2.3. Determination of the MIC of RA Against S. agalactiae ATCC 27956

RA powder (1 g, Sigma-Aldrich, St. Louis, MO, USA) was dissolved in 40 mL DMSO (Solarbio Science & Technology Co., Ltd., Beijing, China), and then added to pre-sterilized Todd-Hewitt broth (THB; Hope Bio-Technology, Qingdao, China) at the required concentration. The stock solution was incorporated into pre-sterilized culture media to achieve target concentrations. Medium without RA (0 mg/mL) served as the control. Bacterial (*S. agalactiae* ATCC 27956) suspensions were adjusted to 1 × 10^5^ CFU/mL via serial two-fold dilution. Replicate wells of a 96-well plate (Baidi Biotech Co., Ltd., Hangzhou, China, Cat. No. H803004) were loaded with 100 μL of bacterial suspension and 100 μL of THB containing RA to yield final concentrations of 0.125, 0.25, 0.5, 1, 2, 3, 4, and 8 mg/mL (final volume: 200 μL/well; *n* = 5 per concentration) [23]. Plates were incubated at 37 °C for 24 h. The MIC was defined as the lowest drug concentration yielding an OD_600_ difference of ≤0.05 compared to sterile negative controls [24].

### 2.4. Determination of Bacterial Adhesion Capacity

Bovine mammary epithelial cells (MAC-T), at passage 5, were cultured to form dense monolayers in T25 cell culture flasks; harvest viable cells by centrifugation at 550× *g* for 5 min. Discard the supernatant and resuspend cells in complete culture medium. The cell suspension was added into a 96-well cell plate at 200 μL per well (≈1 × 10^4^ cells/well). The cells were incubated at 37 °C with 5% CO_2_ until use.

Based on research conducted by Ekambaram et al., RA solutions were prepared at serial concentrations (0, 2, 4, 6, 8, and 10 mg/mL) via gradient dilution [22]. For test groups: One volume of RA solution was mixed with nine volumes of bacterial suspension (1 × 10^8^ CFU/mL *S. agalactiae*). For the control group: Combine 1 volume of culture medium with 9 volumes of bacterial suspension. Co-culture all tubes at 37 °C with shaking (180 rpm) for 18 h. Dilute co-cultured bacteria to 1 × 10^5^ CFU/mL in antibiotic-free complete medium. Store at 4 °C.

When MAC-T cells in the 96-well plate reach more than 90% confluence (about 3 × 10^4^ cells), aspirate the cell culture medium, rinse twice with PBS, add 100 μL of diluted bacterial solution (10^5^ CFU/mL) to each well, and continue culturing under the original culture conditions. Added 100 μL of sterile medium to control wells. All wells were prepared in triplicate. Added 100 μL of THB containing RA to achieve final RA concentration gradients of 0, 2, 4, 6, 8, and 10 mg/mL in a final 200 μL reaction volume per well of the 96-well plate. Samples were collected 2.5 h after bacterial inoculation to determine the adhesion ability of *S. agalactiae* to MAC-T cells. Weighed 3.7 g of brain heart infusion medium (Qingdao Hope Bio-Technology Co., Ltd., Qingdao, China), dissolved it in 100 mL of ultrapure water, then sterilized it by autoclaving at 121 °C for 20 min, and cooled it to room temperature for later use. Collected MAC-T cells together with *S*. *agalactiae* adhered to the cells, added 1 mL of 1% Triton X-100 (*v*/*v*) (Solarbio Science & Technology Co., Ltd., Beijing, China) to each well, and let it stand at room temperature for 10 min; after the cells lysed and the bacteria were released, pipetted to mix evenly, and diluted the suspension with PBS (Solarbio Science & Technology Co., Ltd., Beijing, China) in a 1:1 ratio. Each concentration was tested in three wells. Aspirated 100 μL of the above diluent, inoculated it onto brain heart infusion solid medium, placed it in a 37 °C constant temperature incubator for 48–72 h, took it out for colony counting, and calculated the total number of *S. agalactiae* in each well.

### 2.5. Inhibitory Effects of RA on S. agalactiae Biofilm Formation

*S. agalactiae* was cultured in THB overnight (37 °C, 180 rpm), diluted to OD_600_ = 0.05, and dispensed into 96-well plates (100 μL/well) with 100 μL of RA (0, 2, 4, 6, 8, and 10 mg/mL; 6 wells per group). After 24 h incubation at 37 °C, wells were rinsed three times with PBS, air-dried, stained with 0.1% crystal violet for 15 min, and washed. Dye was solubilized in 200 μL of 95% ethanol, and absorbance was measured at OD_590_.

*S. agalactiae* was cultured overnight in THB in the presence or absence of RA at its MIC, serving as the experimental and control groups, respectively. Bacterial cells were harvested by centrifugation at 500× *g* for 5 min at 4 °C, yielding a pellet volume of 20–30 mm^3^. After discarding the supernatant, the pellets were fixed in 2.5% glutaraldehyde solution. The bacterial aggregates were gently resuspended using a sterile toothpick, fixed for at least 4 h, and stored at 4 °C. Subsequently, the samples were centrifuged at 500× *g* for 5 min, the supernatant was removed, and 0.1 M phosphate buffer (pH 7.4) was added. Following gentle mixing and a 3 min rinse, the suspension was centrifuged; this washing cycle was repeated three times. A 1% (*w*/*v*) agarose solution was prepared by heating until fully dissolved and dispensed into microcentrifuge tubes after cooling slightly. Prior to agarose gelation, the bacterial pellet was carefully isolated with forceps, suspended, and embedded within the agarose matrix. The embedded specimens were rinsed three times with 0.1 M phosphate buffer (pH 7.4) for 15 min per wash, followed by post-fixation with 1% osmium tetroxide diluted in 0.1 M phosphate buffer (pH 7.4) for 2 h at room temperature. Finally, the samples were washed three times with 0.1 M phosphate buffer (pH 7.4) for 15 min each. Specimens were dehydrated through a graded ethanol series (30%, 50%, 70%, 80%, 85%, 90%, and 100% *v*/*v*) for 15–20 min per step, with the 100% ethanol step performed in duplicate. For specimens requiring extended dehydration, incubation times were adjusted accordingly. Subsequently, the samples were incubated twice in 100% acetone for 15 min per cycle as a transitional solvent Resin infiltration was carried out sequentially using mixtures of acetone and SPI-Pon™ 812 epoxy resin (Structure Probe, Inc., West Chester, PA, USA) under the following conditions: acetone to SPI-Pon™ 812 at a 1:1 (*v*/*v*) ratio for 2–4 h at 37 °C; acetone to SPI-Pon™ 812 at a 1:2 (*v*/*v*) ratio overnight at 37 °C; and pure SPI-Pon™ 812 resin for 5–8 h at 37 °C. The specimens were then transferred into embedding molds filled with fresh SPI-Pon™ 812 resin and pre-incubated at 37 °C overnight. Finally, the molds were transferred to a 60 °C oven for polymerization over 48 h to yield solid resin blocks. Ultrathin sections (60–80 nm thick) were cut using a Leica UC7 ultramicrotome and mounted on 150-mesh Formvar-coated copper grids (Leica Microsystems IR GmbH, Wetzlar, Germany). The sections were stained with 2% saturated ethanolic uranyl acetate in the dark for 8 min, rinsed three times with 70% ethanol, and washed three times with ultrapure water. Post-staining was performed using 2.6% lead citrate solution in a CO_2_-free environment for 8 min, followed by three rinses with ultrapure water. Grids were blotted gently with filter paper and dried overnight in a grid box at room temperature. Ultrastructural examination and image acquisition were conducted using a transmission electron microscope TECNAI G 20 TWIN (FEI Company, Hillsboro, OR, USA).

### 2.6. Establishment of a Postpartum/Lactating Mouse Model with S. agalactiae-Induced Mastitis

Female Kunming mice 8–10 days postpartum were divided into a sterile normal saline group (*n* = 6, biological replicates), bacterial challenge group (*n* = 6, biological replicates), bacterial challenge + 25 mg/kg drug injection group (*n* = 6, biological replicates), bacterial challenge + 50 mg/kg drug injection group (*n* = 6, biological replicates), and bacterial challenge + 100 mg/kg drug injection group (*n* = 6, biological replicates). One hour before the experiment, pups were separated from the dam to allow mammary gland engorgement. In the control group, 50 μL of sterile normal saline was injected into the nipple duct of the 4th pair of mammary glands; in the bacterial challenge group, 50 μL of *S. agalactiae* suspension (5 × 10^4^ CFU) was injected into the nipple duct of the 4th pair of mammary glands for 24 h. In the bacterial challenge + drug injection groups, 50 μL of *S. agalactiae* suspension (5 × 10^4^ CFU) was injected into the nipple duct of the 4th pair of mammary glands. After bacterial challenge, the drug was dissolved in normal saline. Intraperitoneal injections were performed at 6, 12, 18, and 24 h post-challenge. The single-injection doses for each group was 6.25 mg/kg, 12.5 mg/kg, and 25 mg/kg, respectively, resulting in cumulative 24 h doses of 25 mg/kg, 50 mg/kg, and 100 mg/kg after four administrations, followed by collection of samples from the 4th pair of mammary glands, which is according to the search of Jiang et al. [22]. After injecting 2 IU of oxytocin into each dam, approximately 20 μL of milk was collected from the 4th pair of mammary glands using a self-made oral pipette for white blood cell counting. After a 4 h fasting period, mice were transferred to clean containers and gradually exposed to carbon dioxide. The mice were anesthetized via exposure to 40% CO_2_ for 10 min to achieve painless euthanasia. If any mice exhibited vital signs following this procedure, cervical dislocation was conducted as a supplementary measure. Approximately 1 mL of blood was collected from each dam via orbital puncture for routine blood detection and analysis.

### 2.7. Pathological Structures of Mice Mammary Gland Were Examined by Hematoxylin-Eosin (HE) Staining

Tissue samples measuring (2 × 2 × 2 mm) were harvested and fixed in 4% paraformaldehyde for 48 h. The fixed tissues were rinsed with running water to remove fixative and impurities. Specimens were dehydrated through a graded ethanol series (50%, 70%, 85%, 95%, and 100% *v*/*v*) for 2 h per concentration. Clearing was performed by immersing the samples in a 1:1 (*v*/*v*) mixture of ethanol and xylene for 2 h, followed by two successive incubations in pure xylene for 2 h each. Subsequently, tissues were infiltrated with a 1:1 (*v*/*v*) mixture of molten paraffin and xylene for 1–2 h, followed by two changes in pure molten paraffin for approximately 3 h each. The infiltrated tissues were embedded in molten paraffin within molds and oriented appropriately. Upon solidification, excess paraffin was trimmed, and sections (4–5 μm thick) were cut using a Leica microtome (Leica Microsystems IR GmbH, Wetzlar, Germany).

Paraffin sections were deparaffinized in two changes of xylene for 20 min each, then rehydrated through a graded ethanol series (100%, 100%, and 75% *v/v* for 5 min per step) and rinsed in distilled water for 3 min. Sections were stained with hematoxylin for 3–5 min and washed under running tap water to remove excess stain. Differentiation was performed in 1% (*v*/*v*) hydrochloric acid aqueous solution for several seconds, followed by rinsing under tap water. The sections were blued in a 0.6–0.7% (*v*/*v*) aqueous ammonia solution and washed briefly under running tap water. Following partial dehydration through 85% and 95% ethanol, sections were counterstained with eosin for 5 min. Subsequently, sections were fully dehydrated in three changes of 100% ethanol (5 min each), cleared in n-butanol for 5 min, followed by xylene for 5 min, and mounted with neutral balsam. Histological examination and image acquisition were conducted using a Leica DM1000 microscope equipped with the Leica Application Suite imaging system (Leica Microsystems IR GmbH, Wetzlar, Germany).

### 2.8. The Levels of Pro-Inflammatory and Anti-Inflammatory Cytokines Were Determined Using ELISA

For mammary tissue samples, weigh 20 mg of dissected tissue, add 200 μL of 0.1 M phosphate buffer (pH 7.4), and fully homogenize the sample manually or using a tissue homogenizer. The homogenate was centrifuged at 500× *g* for 20 min, and the supernatant was carefully collected.

ELISA kits were purchased from MEINIAN Co., Ltd. (Yancheng, China) (Supplementary Table S1). First, dilute the standard; then set up blank wells (no sample or enzyme-labeled reagent is added to the blank control wells, while all other steps remain the same), standard wells, and test sample wells, respectively. Accurately add 50 μL of the standard to the enzyme-labeled coated plate. For the test sample wells, first add 40 μL of sample dilution, then add 10 μL of the test sample (the final dilution of the sample is 5-fold). Sealed the plate with a sealing film and incubated at 37 °C for 30 min. Discarded the liquid, added washing solution, let it stand for 30 s, then discarded the liquid. Repeated this washing process 5 times and finally patted dry. Added 50 μL of enzyme labeling solution to each well, except for the blank wells. Incubated at 37 °C for 30 min. After repeating the washing process 5 times, add the chromogenic agent, gently shake to mix well, and incubate at 37 °C in the dark for 10 min. Add 50 μL of stop solution to terminate the reaction. Each sample was repeated in 3 wells. Zero the instrument with the blank well, and measure the absorbance (OD value) of each well in sequence at a wavelength of 450 nm. The measurement should be carried out within 15 min after adding the stop solution.

### 2.9. The Expression of Inflammatory Factors and Related Genes Was Quantified by RT-qPCR

*S. agalactiae* was cultured overnight in THB with/without RA at MIC (test/control groups). Cultures were centrifuged at 8000× *g* for 2 min, washed 3 times with 0.1 M phosphate buffer (pH 7.4), collected the bacteria, added lysozyme, incubated at 37 °C for 5 min, then centrifuged at 8000× *g* for 2 min to collect the precipitate for bacterial RNA extraction; add liquid nitrogen to 1 mg of mouse mammary gland tissue, grind it, and use it for total tissue RNA extraction. The A260/A280 ratio of RNA was in the range of 1.9–2.2. After determining the total RNA concentration, cDNA was synthesized according to EasyScript^®^ One-Step gDNA Removal and cDNA Synthesis SuperMix (TransGen Biotech, Beijing, China). Specific primers were designed based on the sequences in NCBI, and the primer sequence information is shown in Supplementary Table S2, which was synthesized by Qingke Biotechnology Co., Ltd. (Beijing, China). The relative expression level of target gene mRNA was detected by RT-qPCR (RocGene (Beijing, China) Technology Co., Ltd., Beijing, China, Mod. No. Archimed-X4). The reaction volume was 20 μL: 10 μL of TransStart^®^ Tip Green qPCR SuperMix (TransGen Biotech, Beijing, China), 0.5 μL of each primer, 1 μL of cDNA, and 8 μL of ddH_2_O. The reaction program was as follows: 95 °C for 30 s, 95 °C for 5 s, 60 °C for 30 s, with a total of 40 cycles. Each sample was repeated in 3 wells. After the reaction, the amplification curve and melting curve were observed, and the value of Ct was calculated. *GAPDH* and *16S* genes were used as the internal reference genes for the normalization of relative gene expression levels. For the experiment, 3 independent biological replicates were performed, with 3 technical replicates per biological replicate. The 2^−△△Ct^ method was used to calculate the relative expression level of the target gene mRNA.

### 2.10. Protein Levels of Related Signaling Pathways Were Determined by Western Blotting

20. mg of mammary gland tissue was ground with liquid nitrogen, then 1 mL of RIPA lysis buffer (Solarbio Science & Technology Co., Ltd., Beijing, China), protease inhibitor (Solarbio Science & Technology Co., Ltd., Beijing, China), and protein phosphatase inhibitor (Solarbio Science & Technology Co., Ltd., Beijing, China) were added. The mixture was lysed at 4 °C for 30 min, and then centrifuged at 8000× *g*/min at 4 °C for 30 min. The supernatant was gently aspirated into a new tube and stored at −80 °C for later use. The total protein concentration was determined using a BCA kit (Solarbio Science & Technology Co., Ltd., Beijing, China). After quantification, the protein was mixed with protein loading buffer, denatured in a metal bath at 95 °C for 5 min, and stored at −20 °C for later use. Denatured protein samples (10 μg/lane) were resolved by SDS-PAGE (5% stacking, 12% resolving) and transferred onto PVDF membranes (Millipore, Boston, MA, USA). Membranes were blocked with 5% skimmed milk in 1 × TBST (Solarbio Science & Technology Co., Ltd., Beijing, China) for 2.5 h at room temperature, incubated with primary antibodies (Supplementary Table S3) overnight at 4 °C, and probed with HRP-conjugated secondary antibodies (1/5000) (Biosynthesis Bio. Ltd., Beijing, China) for 1.5 h at 37 °C. Take equal volumes of Meilunbio^®^ Femtochem Super-Sensitive ECL Luminescent Solution A and B (Meilunbio, Dalian, China), mix well, apply the mixture onto the front side of the membrane, and incubate for 5 min in the dark. Scanned and analyzed the results using a chemiluminescence imaging system SCG-W3000 (Wuhan Servicebio Technology Co., Ltd., Wuhan, China). The grayscale values were analyzed using Image-J software (Version 1.7).

### 2.11. Statistical Analysis

SPSS 24.0 (IBM, Armonk, New York, USA) and GraphPad Prism (GraphPad Software version 10.0, Boston, MA, USA) were used to analyze the data, which were expressed as “mean ± standard deviation”. The independent *t*-test was adopted for comparisons between only two groups. One-way ANOVA was used for multiple-group comparisons, followed by Tukey’s post hoc test for all pairwise comparisons. Normality was assessed by the Shapiro–Wilk test, and Levene’s test was used to evaluate homogeneity of variance. n corresponds to independent biological replicates. *p* < 0.05 indicated a significant difference, and *p* < 0.01 indicated an extremely significant difference.

## 3. Results

### 3.1. The Effect of RA on S. agalactiae

#### 3.1.1. Effects of RA on Functional and Structural Characteristics of *S. agalactiae*

**Figure 1:**
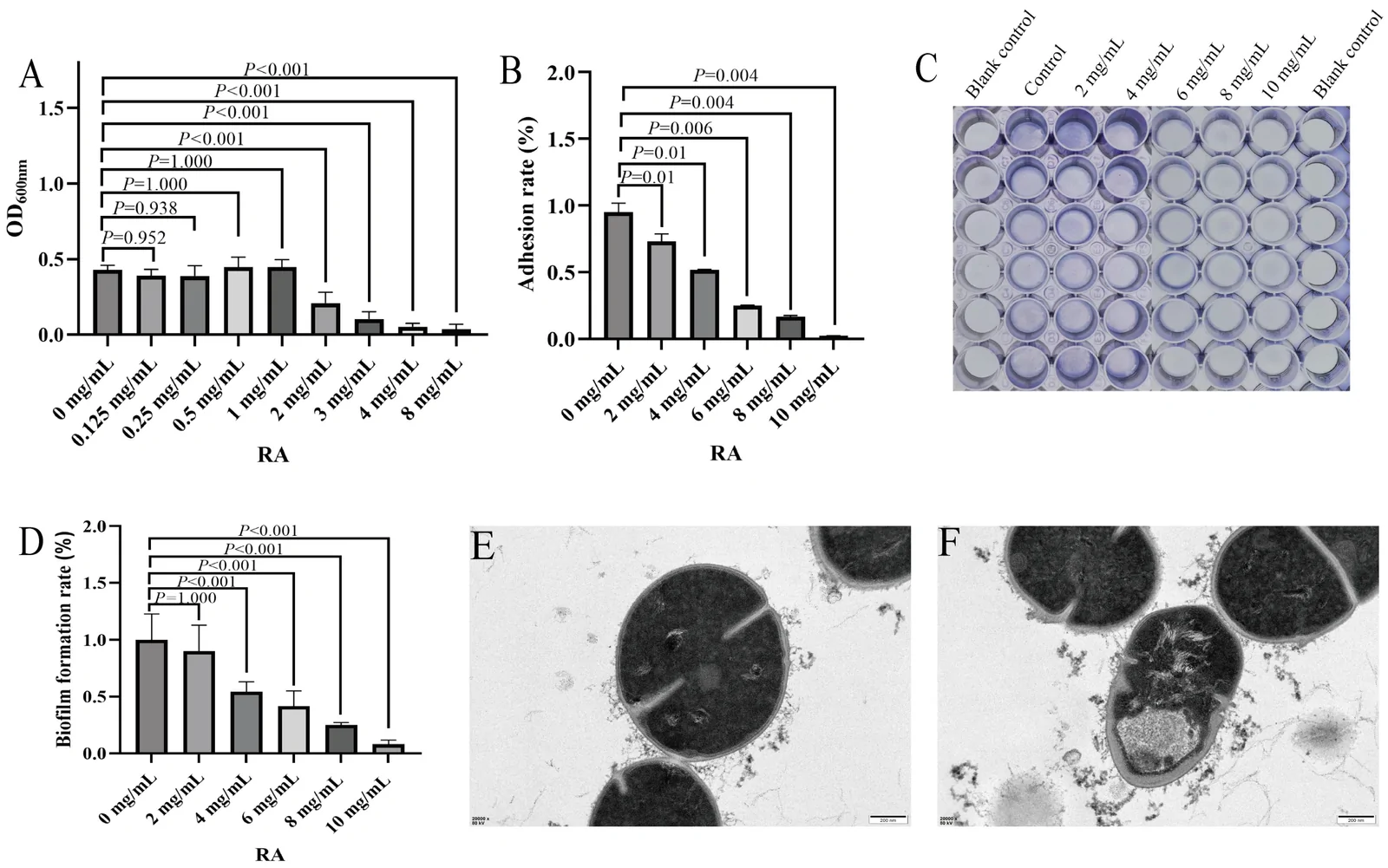
The antibacterial effect of RA against *S. agalactiae*. (**A**) The MIC of RA against *S. agalactiae*; For the experiment, 3 independent biological replicates were performed, with 3 technical replicates per biological replicate; One-way ANOVA followed by Tukey’s HSD test. (**B**) The effect of RA on the adhesion rate of *S. agalactiae* standard strain to mammary epithelial cells; For the experiment, 3 independent biological replicates were performed, with 3 technical replicates per biological replicate; One-way ANOVA followed by Tukey’s HSD test. (**C**) The effect of RA on biofilm formation of the *S. agalactiae* standard strain; (**D**) Inhibition rate of biofilm formation of *S. agalactiae* by RA. For the experiment, 3 independent biological replicates were performed, with 3 technical replicates per biological replicate; One-way ANOVA followed by Tukey’s HSD test. (**E**) *S. agalactiae* under TEM; (**F**) The effect of RA at MIC concentration on capsular production of *S. agalactiae* under TEM.

*S. agalactiae* was co-cultured with different concentrations of RA for 24 h to determine the minimum inhibitory concentration (MIC). When the RA concentration was 0.125, 0.25, 0.5, or 1 mg/mL, it had little effect on the proliferation of *S. agalactiae*, with no significant difference observed compared with the control group (0 mg/mL) (*p* > 0.05). At an RA concentration of 2 mg/mL, the proliferative capacity of *S. agalactiae* decreased significantly compared with that of the control group (*p* < 0.05). When the RA concentration reached 3, 4, and 8 mg/mL, the proliferative capacity of *S. agalactiae* decreased further, showing highly significant differences compared with the control group (*p* < 0.01) (Figure 1A), indicating that RA exhibited potential antibacterial activity.

Bacterial suspensions of *S. agalactiae* cultured with different concentrations of RA were inoculated onto a monolayer of mammary epithelial cells to evaluate the adhesion of *S. agalactiae* to these cells. The results are shown in Figure 1B. Compared with the control group (0 mg/mL), treatment with 2 mg/mL RA reduced the adhesion rate of *S. agalactiae* to mammary epithelial cells to 73.04% (*p* < 0.05). At an RA concentration of 4 mg/mL, the adhesion rate was significantly reduced (*p* < 0.05). When the RA concentration reached 6 mg/mL or higher, the adhesion rate of *S. agalactiae* to mammary epithelial cells was further reduced, with highly significant differences compared with the control group (*p* < 0.01). At the MIC concentration, the adhesion capacity of *S. agalactiae* decreased to 16.6%, which was significantly lower than that of the control group (*p* < 0.01).

Compared with the control group (0 mg/mL), biofilm formation by *S. agalactiae* decreased to 90.09% following treatment with 2 mg/mL RA, with no significant difference observed (*p* > 0.05). Results of crystal violet staining indicated that elevated concentrations of RA suppressed biofilm formation by *S. agalactiae* (Figure 1C). When the RA concentrations were 4, 6, and 8 mg/mL, biofilm formation by *S. agalactiae* was further inhibited, decreasing to 54.48%, 41.78%, and 25.09%, respectively, with highly significant differences (*p* < 0.01) (Figure 1C,D). Compared with the control group, 10 mg/mL RA decreased *S. agalactiae* biofilm formation to 8.13% (*p* < 0.01).

To further visualize the effect of RA on bacterial morphology, *S. agalactiae* was treated with RA at the minimum inhibitory concentration (MIC, 8 mg/mL) and examined by transmission electron microscopy (TEM) after overnight incubation. Changes in bacterial ultrastructure following RA treatment were clearly observed. As shown in Figure 1E, untreated bacteria exhibited intact cellular morphology with no obvious structural damage. In contrast, RA-treated bacteria (Figure 1F) showed marked structural damage, with some cells ruptured and exhibiting leakage of intracellular contents, whereas others appeared deformed and wrinkled.

#### 3.1.2. Effects of RA on Virulence Factors of *S. agalactiae*

**Figure 2:**
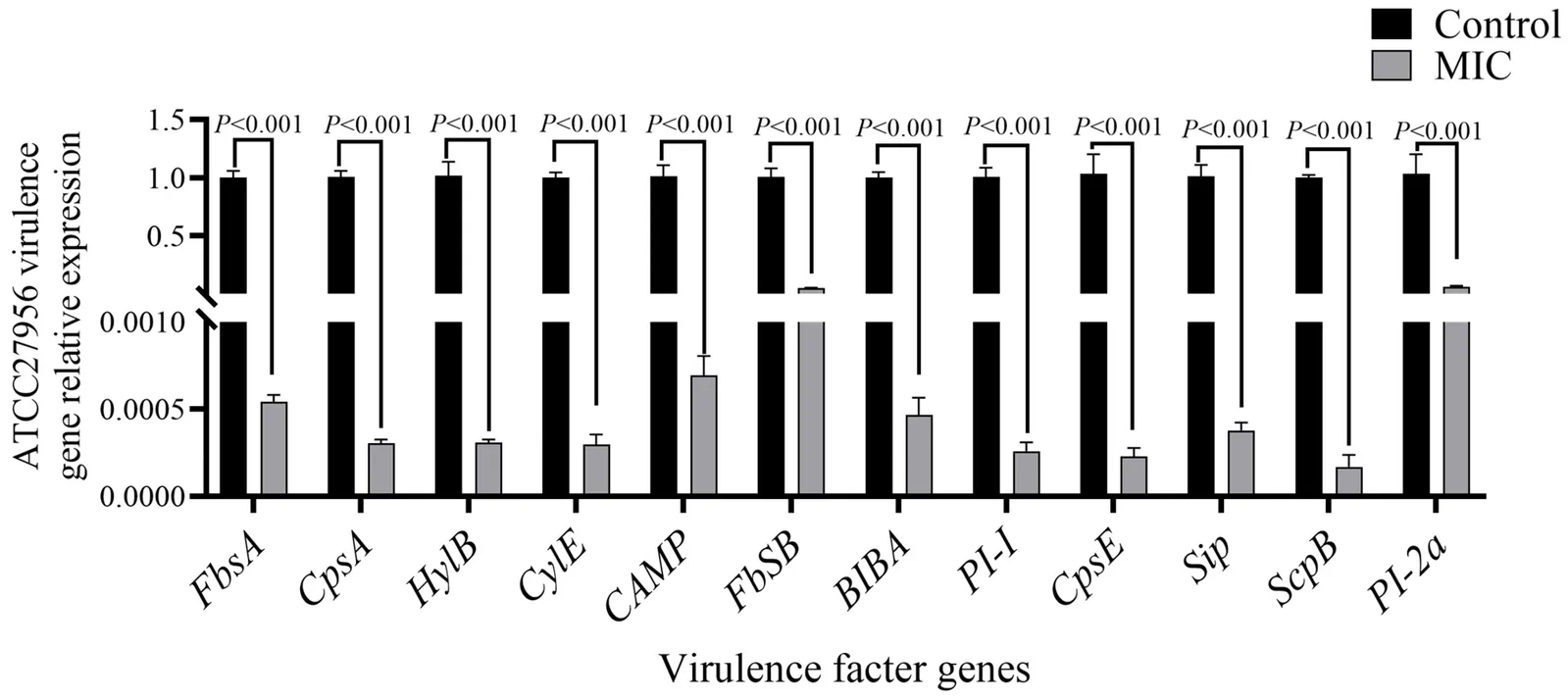
The effect of RA at MIC (8 mg/mL) on the expression of virulence genes in *S. agalactiae*. 3 independent biological replicates were performed, with 3 technical replicates per biological replicate; Independent *t*-test.

RT-qPCR results revealed that after RA treatment, the expression levels of genes associated with invasion and immune evasion, including *ScpB*, *CpsA*, *CylE*, *HylB*, and *CAMP*, were significantly decreased compared to the control group (*p* < 0.01) (Figure 2). Genes related to capsular polysaccharide synthesis, such as *CpsE*, were likewise significantly downregulated (*p* < 0.01). Additionally, the expression levels of adhesion-related genes (*BIBA*, *PI-2a*, *Sip*, *FbsA*, and *FbsB*) were significantly reduced in the RA-treated group (*p* < 0.01). The transcription of pilus-protein-encoding *PI-1* was significantly decreased in the RA-treated group (*p* < 0.01).

### 3.2. Anti-Inflammatory Effects of RA in S. agalactiae-Infected Postpartum/Lactating Mice

#### 3.2.1. Somatic Cell Count in Milk of Postpartum/Lactating Mice

As shown in Table 1, the somatic cell count in the model group was significantly higher than that in the control group and all treatment groups (*p* < 0.05). Notably, no significant difference was observed between the 25 mg/kg and 50 mg/kg treatment groups (*p* > 0.05), whereas the 100 mg/kg treatment group showed no statistically significant difference compared with the control group (*p* > 0.05).

#### 3.2.2. Detection of Inflammatory Factor Expression by RT-qPCR

**Figure 3:**
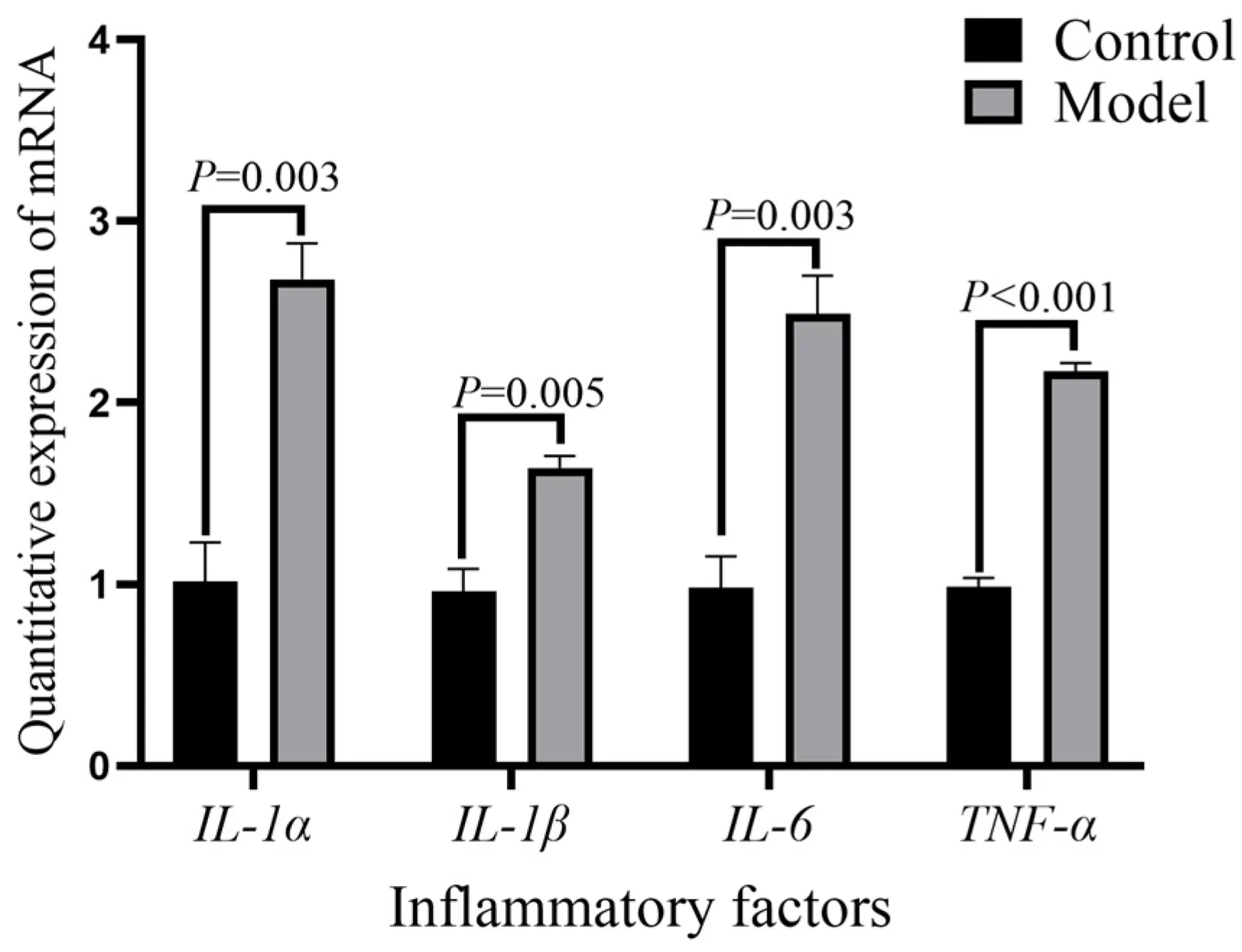
Expression of inflammatory factors in a model of mastitis in postpartum/lactating mice mammary gland infected with *S. agalactiae*. 6 independent biological replicates were performed, with 3 technical replicates per biological replicate; Independent *t*-test.

To preliminarily verify the establishment of a mouse mastitis model induced by *S. agalactiae*, we detected the expression of *IL-1α*, *IL-1β*, *IL-6*, and *TNF-α* using RT-qPCR. According to the quantitative results (Figure 3), the levels of *IL-1α*, *IL-1β*, *IL-6*, and *TNF-α* in the model group were significantly higher than those in the control group (*p* < 0.05). The results revealed that inflammatory responses occurred in the mammary tissues of mice following *S. agalactiae* induction.

### 3.3. Histopathological Examination of Mammary Gland Tissue in Postpartum/Lactating Mice

**Figure 4:**
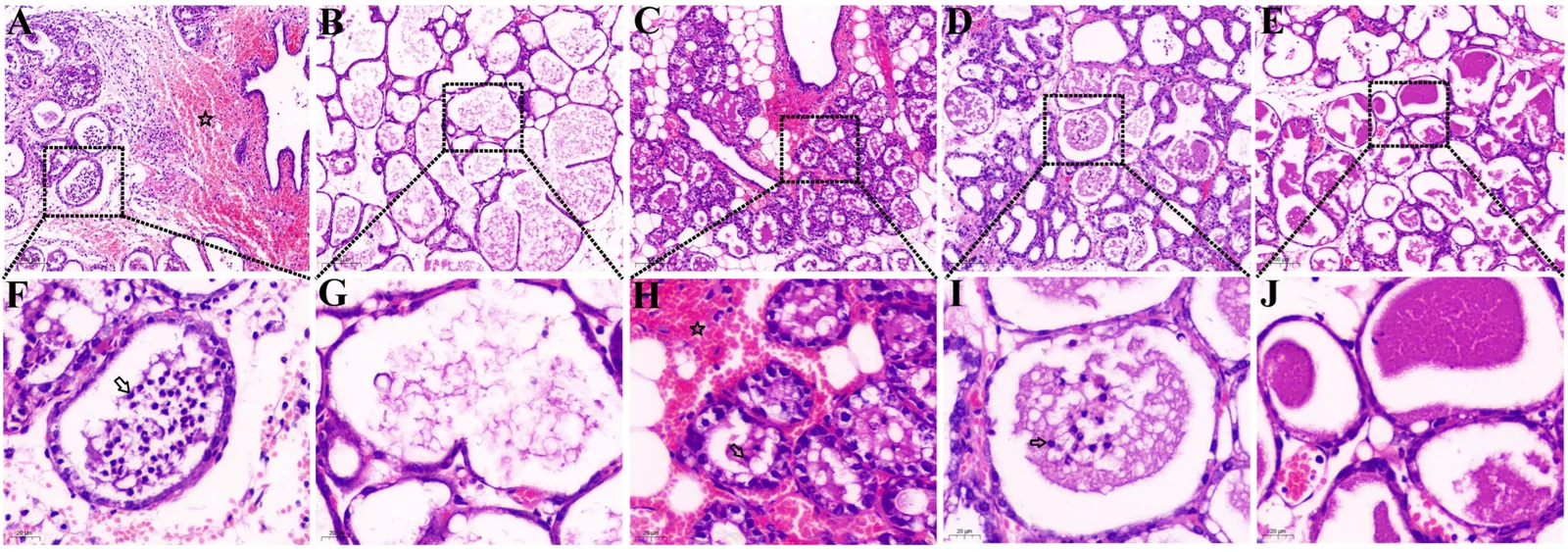
The effect of RA on inflammatory response in mammary glands of postpartum/lactating mice; (**A**) Model group (100×); (**B**) Blank control group (100×); (**C**) 25 mg/kg treatment group (100×); (**D**) 50 mg/kg treatment group (100×); (**E**) 100 mg/kg treatment group (100×); (**F**) Model group (200×); (**G**) Blank control group (200×); (**H**) 25 mg/kg treatment group (200×); (**I**) 50 mg/kg treatment group (200×); (**J**) 100 mg/kg treatment group (200×); The arrow points to shed mammary endothelial cells. The asterisk marks abundant red blood cells within the mammary gland.

Histopathological examination revealed that the model group exhibited thickened ductal walls, extensive exfoliation of epithelial cells into the ductal lumina, and disrupted ductal architecture (Figure 4A,F). In the control group, the mammary ducts exhibited regular, smooth contours with well-organized epithelial cells and intact luminal structures (Figure 4B,G). In the 25 mg/kg treatment group, the ductal walls remained thickened and exfoliated epithelial cells were still present within the lumina; however, the luminal epithelium was relatively more intact than that in the model group (Figure 4C,H). The 50 mg/kg treatment group exhibited intact ductal structures with persistent wall thickening but markedly fewer exfoliated epithelial cells within the lumina (Figure 4D,I). In the 100 mg/kg treatment group, the ductal walls were noticeably thinner, the ductal architecture was fully preserved, and only minimal epithelial cell exfoliation was observed (Figure 4E,J).

### 3.4. Hematological Parameters in RA-Treated Postpartum/Lactating Mice with S. agalactiae Mastitis

Hematological analysis revealed that all blood cell counts in the model group were significantly higher than those in the blank control group (*p* < 0.05) (Table 2). In the 25 mg/kg treatment group, neutrophil counts showed no significant difference compared with the blank control group, whereas lymphocyte, leukocyte, and intermediate cell counts were significantly lower than those in the model group (*p* < 0.05). The 50 mg/kg treatment group exhibited significantly reduced leukocyte, lymphocyte, intermediate cell, and neutrophil counts compared with the model group (*p* < 0.05). In the 100 mg/kg treatment group, blood cell counts approached those of the blank control group, with no statistically significant differences observed compared with the blank control group.

### 3.5. Results of the ELISA Test for Inflammatory Factors

**Figure 5:**
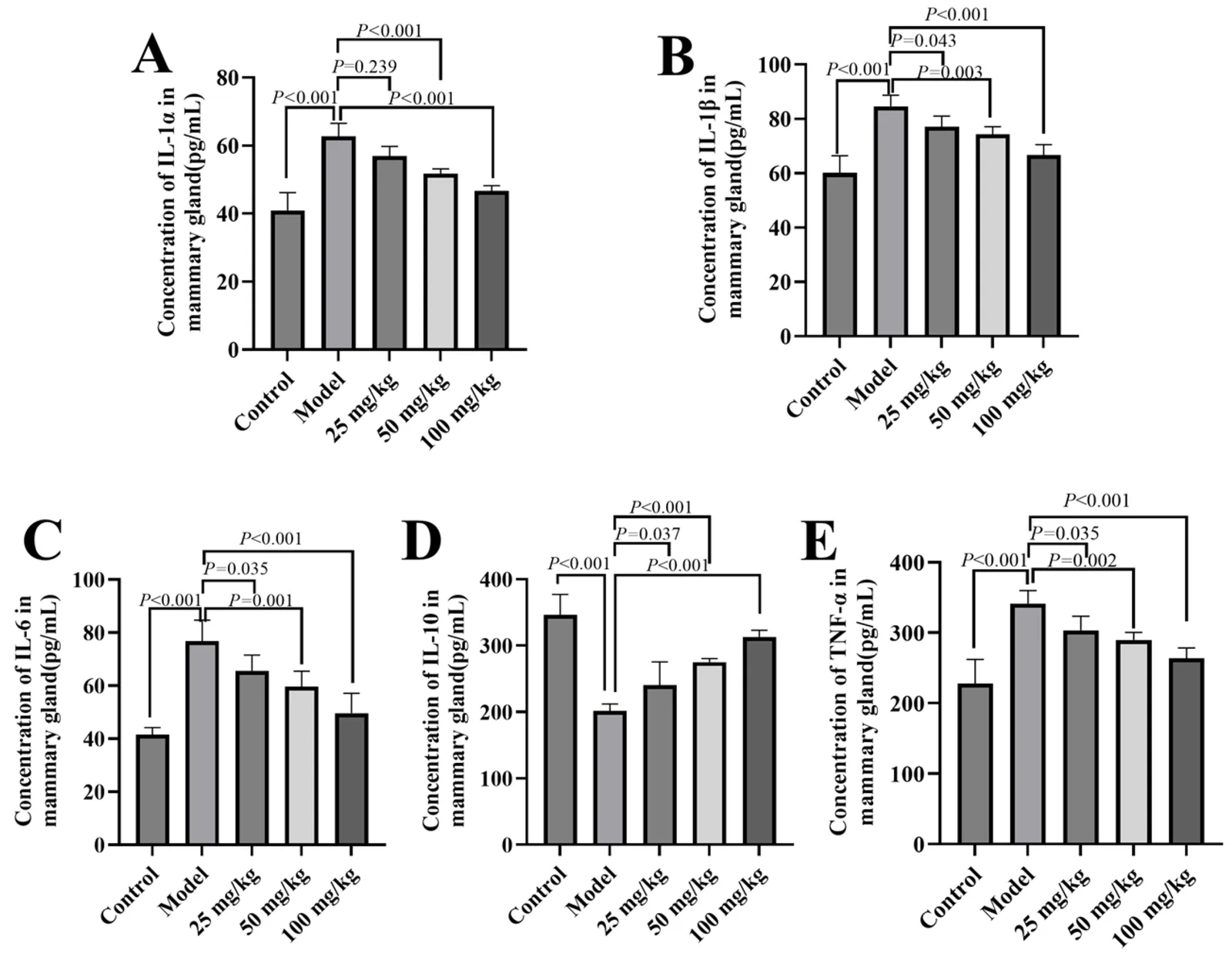
ELISA detection of inflammation-related factor levels in each group. (**A**) The protein level of IL-1α in mammary glands; (**B**) The protein level of IL-1β in mammary glands; (**C**) The protein level of IL-6 in mammary glands; (**D**) The protein level of IL-10 in mammary glands; (**E**) The protein level of TNF-α in mammary glands. 3 independent biological replicates were performed, with 3 technical replicates per biological replicate; One-way ANOVA followed by Tukey’s HSD test.

Compared with the control group, the levels of pro-inflammatory factors IL-1α, IL-1β, IL-6 and TNF-α were significantly increased in the model group (*p* < 0.05) (Figure 5A–C,E). Treatment with different doses of RA significantly reduced the expression levels of these pro-inflammatory cytokines. In contrast, the expression level of the anti-inflammatory cytokine IL-10 was significantly increased in the RA-treated groups compared with the model group (*p* < 0.05) (Figure 5D).

### 3.6. Detection of the Expression Trends of Disease and Inflammation Pathway-Related Genes Regulated by RA via RT-qPCR

**Figure 6:**
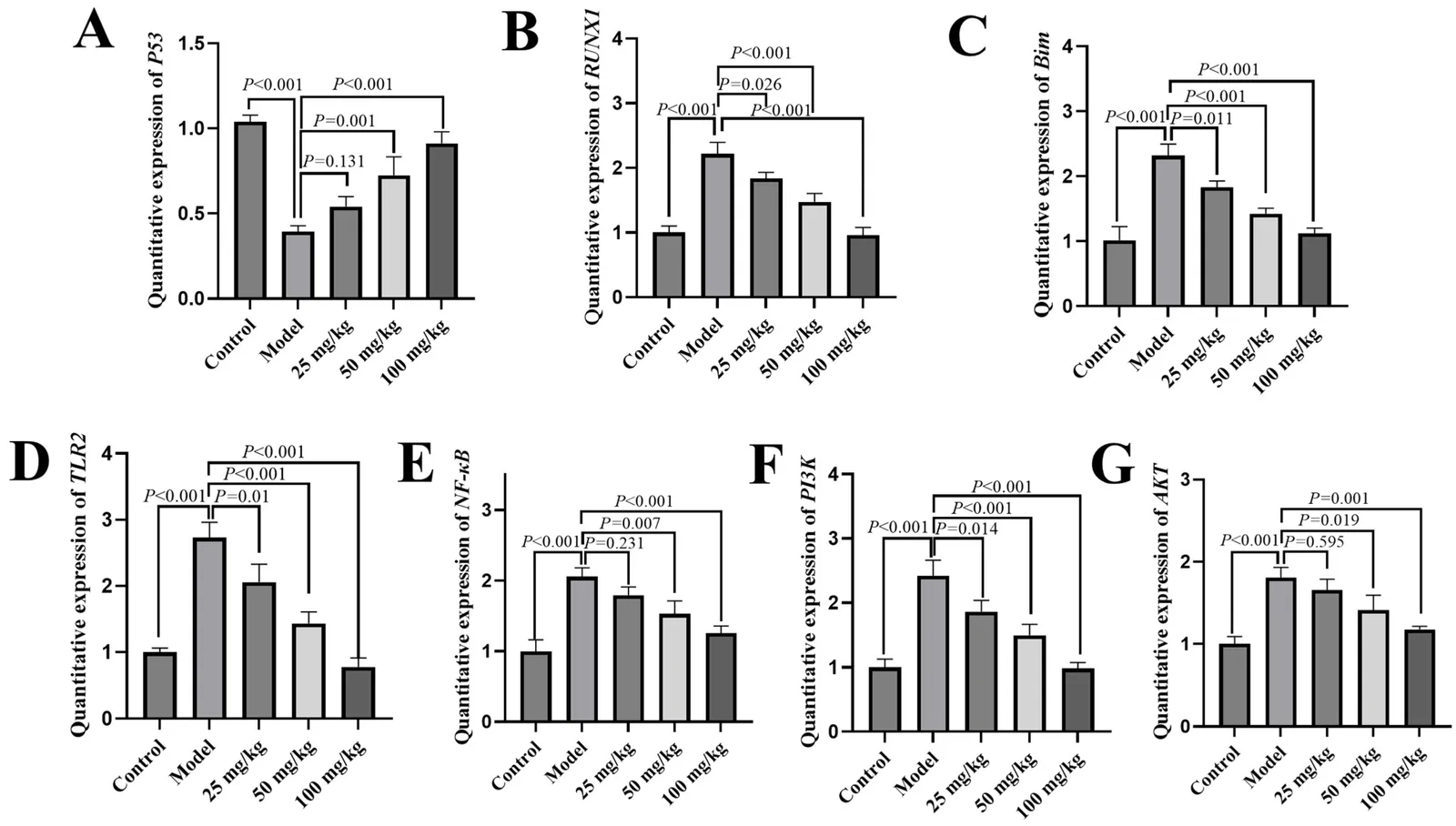
The effect of RA on the expression of genes related to the p53-TLR2/NF-κB pathway in the mammary glands of postpartum/lactating mice. (**A**–**G**) The mRNA expression of *p53*, *RUNX1*, *Bim*, *TLR2*, *NF-κB*, *PI3K*, and *AKT* was determined with RT-qPCR. 3 independent biological replicates were performed, with 3 technical replicates per biological replicate; One-way ANOVA followed by Tukey’s HSD test.

As shown in Figure 6, compared with the control group, the expression level of *p53* in the mammary glands of postpartum/lactating mice infected with *S. agalactiae* was markedly reduced, whereas the expression levels of the other 6 genes (*RUNX1*, *Bim*, *TLR2*, *NF-κB*, *PI3K* and *AKT*) were all significantly upregulated. Following RA treatment of infected postpartum/lactating mice at a dose of 25 mg/kg, the expression levels of *RUNX1*, *Bim*, *TLR2*, and *PI3K* were significantly downregulated (Figure 6B–D,F) (*p* < 0.05). Notably, the expression level of *p53* was significantly upregulated beginning in the 50 mg/kg treatment group (*p* < 0.05) (Figure 6A), whereas the expression levels of *NF-κB* and *AKT* were markedly downregulated (*p* < 0.05) (Figure 6E,G).

### 3.7. Detection of the Effect of RA on the Expression of Disease and Inflammation Pathway-Related Proteins by Western Blotting

**Figure 7:**
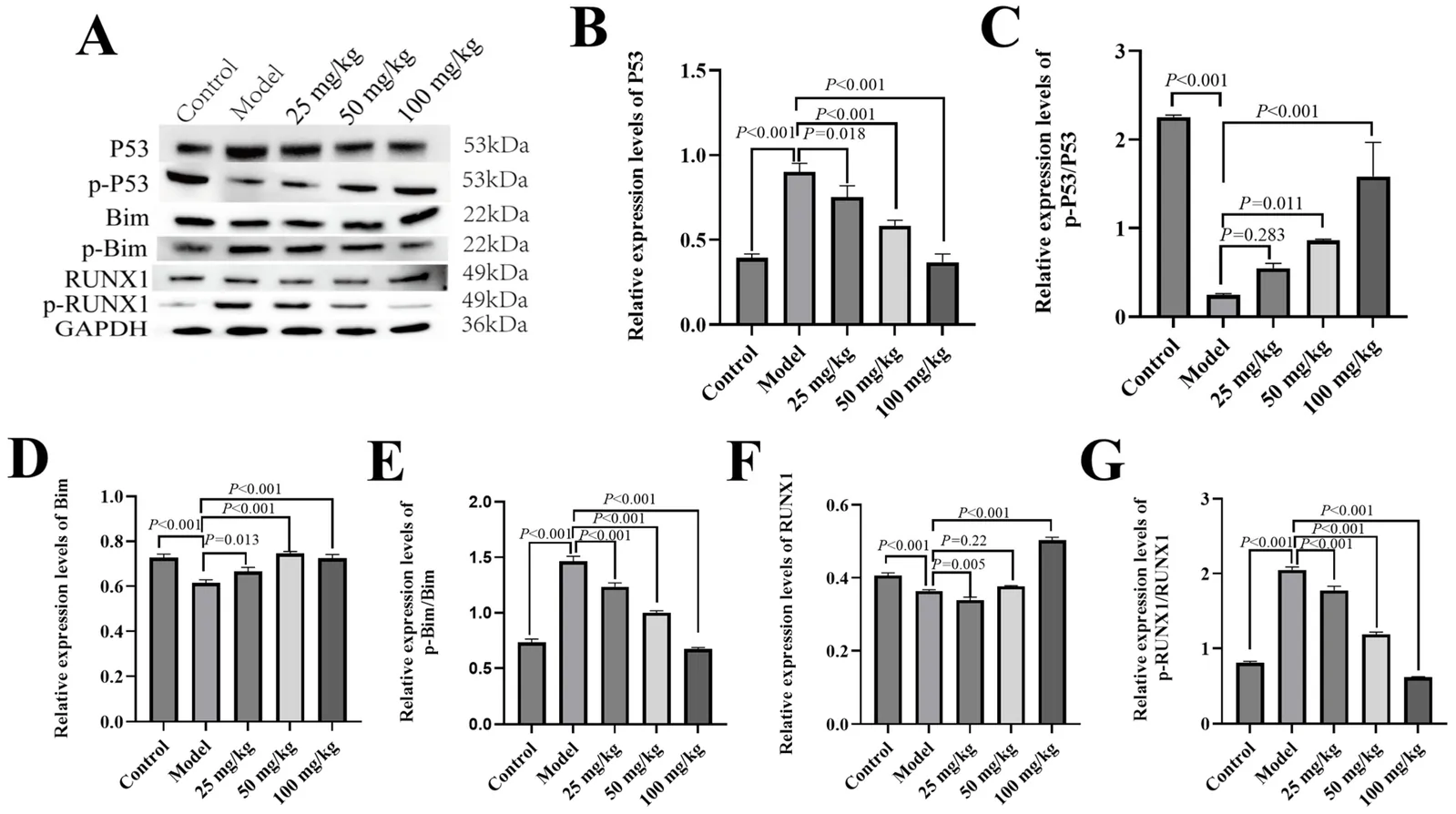
The effect of RA on the p53 pathway in the mammary glands of postpartum/lactating mice. (**A**) Immunoblotting analysis of the protein expression of p53, p-p53, Bim, p-Bim, RUNX1, p-RUNX1. (**B**) The relative levels of p53 are shown as histograms representing density readings of the gel bands, and the ratios were calculated relative to the GAPDH control; (**C**) The ratio of relative levels of p-p53/p53; (**D**) The relative levels of Bim are shown as histograms representing density readings of the gel bands, and the ratios were calculated relative to the GAPDH control; (**E**) The ratio of relative levels of p-Bim/Bim; (**F**) The relative levels of RUNX1 are shown as histograms representing density readings of the gel bands, and the ratios were calculated relative to the GAPDH control; (**G**) The ratio of relative levels of p-Bim/Bim. 3 independent biological replicates were performed, with 3 technical replicates per biological replicate; One-way ANOVA followed by Tukey’s HSD test.

As shown in Figure 7, in postpartum/lactating mice infected with *S. agalactiae*, p53 protein expression was upregulated, whereas it was downregulated following RA treatment. Significant differences were observed between the model group and the RA-treated groups (*p* < 0.05) (Figure 7A,B). Compared with the control group, the phosphorylation level of p53 was decreased in the model group. Following RA treatment, p53 phosphorylation gradually increased, with significant differences observed in the 50 mg/kg and 100 mg/kg treatment groups (*p* < 0.05) (Figure 7A,C). Compared with the control group, Bim protein expression was significantly downregulated in the model group. Following treatment with increasing doses of RA, Bim protein expression gradually increased, although the differences were not statistically significant (*p* > 0.05) (Figure 7A,D). In contrast, the phosphorylation level of Bim was significantly increased in the model group and was significantly reduced following RA treatment (*p* < 0.05) (Figure 7A,E). The expression level of RUNX1 protein in the model group was significantly lower than that in the control group and the 100 mg/kg treatment group (*p* < 0.05) (Figure 7A,F). However, the phosphorylation level of RUNX1 showed the opposite trend, reaching its highest level in the model group and gradually decreasing following RA treatment. The 100 mg/kg treatment group differed significantly from the model group (*p* < 0.05) (Figure 7A,G).

**Figure 8:**
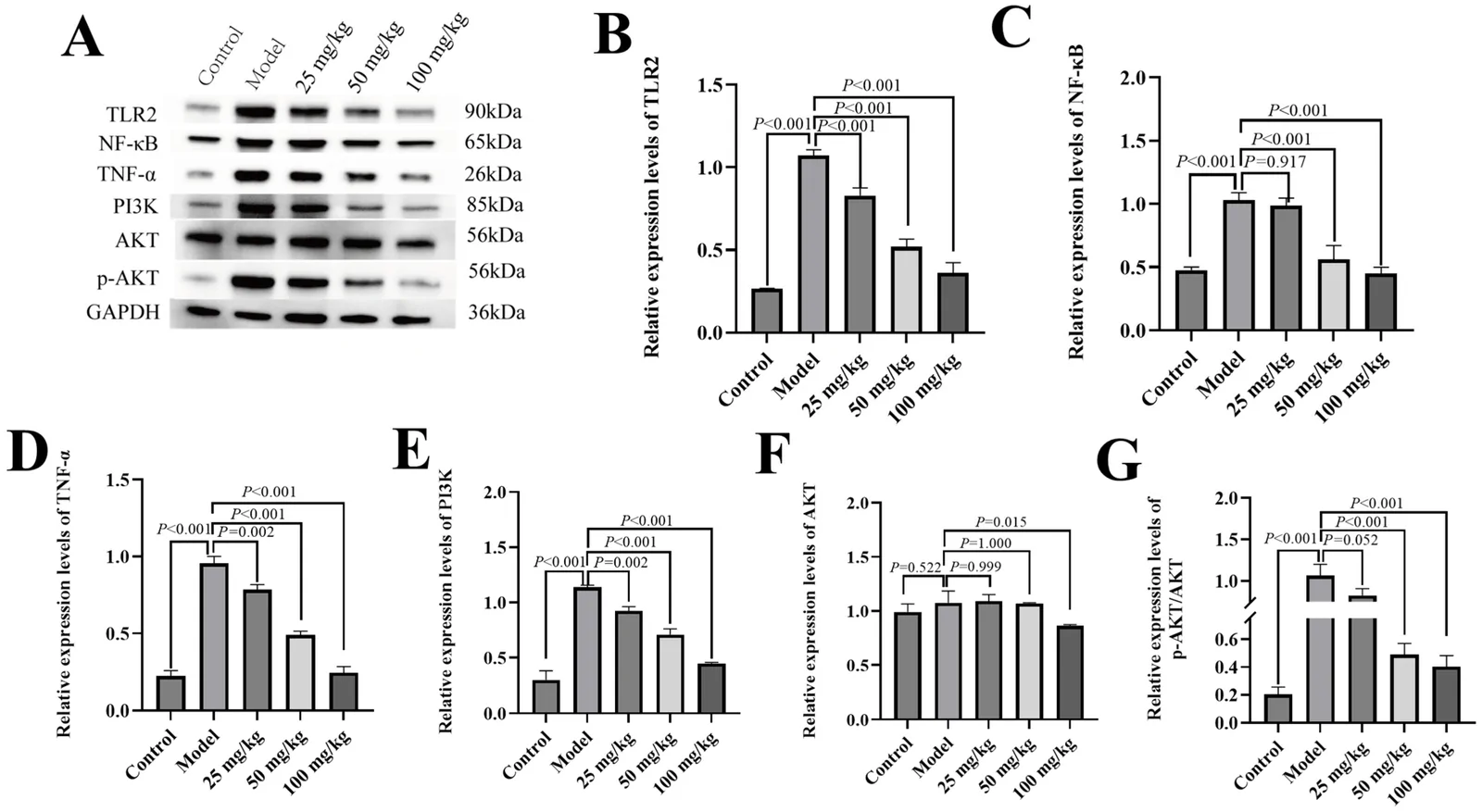
The effect of RA on the TLR2/NF-κB pathway in the mammary glands of postpartum/lactating mice. (**A**) Immunoblotting analysis of the expression of TLR2, NF-κB, TNF-α, PI3K, AKT, p-AKT; (**B**) The relative levels of TLR2 are shown as histograms representing density readings of the gel bands, and the ratios were calculated relative to the GAPDH control; (**C**) The relative levels of NF-κB are shown as histograms representing density readings of the gel bands, and the ratios were calculated relative to the GAPDH control; (**D**) The relative levels of TNF-α are shown as histograms representing density readings of the gel bands, and the ratios were calculated relative to the GAPDH control; (**E**) The relative levels of PI3K are shown as histograms representing density readings of the gel bands, and the ratios were calculated relative to the GAPDH control. (**F**) The relative levels of AKT are shown as histograms representing density readings of the gel bands, and the ratios were calculated relative to the GAPDH control; (**G**) The ratio of relative levels of p-AKT/AKT. 3 independent biological replicates were performed, with 3 technical replicates per biological replicate; One-way ANOVA followed by Tukey’s HSD test.

As shown in Figure 8, compared with the control group, the expression levels of TLR2, NF-κB, TNF-α, PI3K, and p-AKT proteins were significantly upregulated in the model group (*p* < 0.05). Following RA treatment, TLR2 protein expression was progressively downregulated in the treatment groups, with significant differences compared with the model group (*p* < 0.05) (Figure 8A,B). Similarly, NF-κB protein expression was significantly downregulated in the 50 mg/kg and 100 mg/kg treatment groups compared with the model group (*p* < 0.05) (Figure 8A,C). The expression levels of TNF-α and PI3K proteins were also significantly downregulated in the 50 mg/kg and 100 mg/kg treatment groups compared with the model group (*p* < 0.05) (Figure 8A,D,E). No significant difference in AKT protein expression was observed between the control and model groups. However, AKT protein expression was significantly lower in the 50 mg/kg and 100 mg/kg treatment groups than in the model group (*p* < 0.05) (Figure 8A,F). Likewise, p-AKT protein expression gradually decreased with increasing doses of RA and was significantly lower in all RA-treated groups than in the model group (*p* < 0.05) (Figure 8A,G).

## 4. Discussion

Mastitis is a common and costly disease in dairy cows that adversely affects dairy production and causes substantial economic losses. Mastitis-related losses are estimated at $15–45 billion annually in China, and approximately $35 billion globally [25,26], with the monthly incidence of clinical mastitis reaching 3.3% on large-scale dairy farms [27]. Bacterial infection is a major cause of mastitis and can impair mammary gland function, resulting in reduced milk yield and quality. Although antibiotics remain the primary treatment for mastitis, their prolonged and inappropriate use may contribute to antimicrobial resistance and drug residues, raising concerns regarding food safety and public health [20]. Thus, the identification of effective and safer alternatives to conventional antibiotic therapy is of considerable importance. Plant-derived bioactive compounds, particularly polyphenols, have attracted increasing attention because of their potential antibacterial and anti-inflammatory properties and relatively low toxicity [28]. Rosmarinic acid (RA), a major polyphenolic compound found in rosemary, is widely used as a natural antioxidant in food and feed products and has also been reported to possess anti-inflammatory activity [29,30]. However, its potential antibacterial activity against *S*. *agalactiae* and its protective effects against *S. agalactiae*-induced mastitis remain poorly understood. Therefore, the present study evaluated the antibacterial activity of RA against *S. agalactiae* and investigated its anti-inflammatory and protective effects in a mouse model of *S. agalactiae*-induced mastitis.

Previous studies have reported inhibition zones of 30 and 32 mm for 1 mg/mL RA against *S*. *aureus* and *Escherichia coli*, respectively [31]. RA has also been reported to exhibit antiviral activity by reducing mortality and viral load in mice infected with Japanese encephalitis virus [32]. In the present study, the MIC assay identified 8 mg/mL as the MIC of RA against *S. agalactiae*, further supporting its potential antibacterial activity against this pathogen.

Adhesion of *Streptococci* to mammary epithelial cells is a critical step in the early pathogenesis of mastitis [33]. *S. agalactiae* can invade mammary tissue through the lactiferous ducts and adhere to mammary epithelial cells during the initial stage of infection. In the present study, 8 mg/mL RA reduced the adhesion rate of *S. agalactiae* to MAC-T cells to 16.6% and was associated with disruption of bacterial cell membrane integrity. Consistent with previous findings that RA affects biofilm formation in a concentration- and time-dependent manner [34], our results further indicated that RA inhibited the adhesion of *S. agalactiae* to MAC-T cells. This effect may be related to altered expression of virulence genes involved in bacterial adhesion, including *BIBA*, *PI-2a*, *Sip*, *FbsA*, and *FbsB*, although this possibility requires further experimental validation. This interpretation is supported by the findings of Fessia et al., who reported that bacterial adhesion strength correlates with the expression of these virulence genes [35]. Capsular polysaccharide (CPS) is an important virulence factor of *S. agalactiae* that contributes to bacterial invasion and immune evasion [36]. The terminal sialic acid (Sia) residues of CPS can interfere with complement activation, impair phagocytosis, and interact with inhibitory Siglec receptors, thereby suppressing leukocyte function [37,38]. In the present study, 8 mg/mL RA significantly reduced CPS production by *S. agalactiae*. Together with the observed disruption of bacterial membrane integrity, these findings suggest that RA may compromise bacterial virulence and host colonization. However, the precise relationship between RA-induced membrane damage and reduced CPS production remains to be elucidated.

Biofilm formation by *S. agalactiae* on mammary epithelial cells can enhance bacterial survival and reproduction by providing protection against host immune defenses and environmental stresses. In the present study, RA inhibited biofilm formation by *S. agalactiae* in a concentration-dependent manner. Treatment with 2 mg/mL RA reduced biofilm formation to 90.09%, whereas treatment with 4, 6, and 8 mg/mL RA further reduced biofilm formation to 54.48%, 41.78%, and 25.09%, respectively. Furthermore, increasing the RA concentration to 10 mg/mL resulted in a further reduction in *S. agalactiae* biofilm formation, suggesting that RA may interfere with bacterial surface-associated structures and biofilm development. These findings are similar to previous studies demonstrating that tea saponin inhibits *S. agalactiae* biofilm formation by downregulating the *CpsA* gene and reducing the survival rate of *S. agalactiae* [39]. Collectively, it is proposed that RA may influence *S. agalactiae* biofilm formation via the *CpsA* gene. However, further studies are required to elucidate the molecular mechanisms underlying the inhibitory effects of RA on *S. agalactiae* biofilm formation and bacterial persistence within biofilms.

To further investigate the anti-inflammatory effects of RA in *S. agalactiae*-induced mastitis, we established a mouse model of mastitis. Mice were selected as an experimental model because their mammary gland can recapitulate key pathological and inflammatory features of mastitis, and because mice are readily available and amenable to controlled experimental manipulation [40]. Histopathological analysis revealed that RA treatment dose-dependently alleviated the pathological lesions in mammary tissues. The observed pathological changes were consistent with those reported by Zeng et al. in a mouse mastitis model, supporting the successful establishment of the experimental model [41]. Compared with the untreated mastitis group, RA treatment partially restored the structural integrity of the mammary epithelium. These findings suggest that RA may alleviate tissue damage and facilitate the recovery of mammary tissue following *S. agalactiae* infection. Consistent with the histopathological findings, the number of inflammatory cells decreased with increasing RA doses, further supporting the anti-inflammatory effect of RA in the mastitis model.

In addition to its effects on inflammatory responses, the present study further showed that RA attenuated the abnormal apoptosis of mammary epithelial cells induced by *S. agalactiae* infection, suggesting a potential role for RA in maintaining mammary tissue homeostasis [42,43,44,45,46]. IL-10 is an important anti-inflammatory cytokine that contributes to the regulation of excessive inflammatory responses and maintenance of tissue homeostasis [47]. In the present study, *S. agalactiae* infection significantly reduced IL-10 expression in mouse mammary tissues, whereas RA treatment restored IL-10 levels. These findings suggest that RA may enhance the endogenous anti-inflammatory response and alleviate the inflammatory imbalance induced by *S. agalactiae* infection. TNF-α, which is produced predominantly by activated immune cells, is a potent pro-inflammatory cytokine involved in the activation of multiple inflammatory signaling pathways, including the NF-κB pathway [48]. Previous studies have reported that RA can suppress NF-κB activation and TNF-α expression in breast tumors [49]. Consistent with these previous findings, our results showed that RA treatment reduced the expression of TNF-α and other pro-inflammatory mediators and was accompanied by inhibition of NF-κB-related signaling in *S. agalactiae*-induced mastitis. These findings suggest that the anti-inflammatory effects of RA may be associated with modulation of the NF-κB signaling pathway.

Previous studies have shown that the PI3K/AKT and TLR/NF-κB signaling pathways interact to amplify inflammatory responses [50]. Infection with Gram-positive bacteria can activate TLR2, which is involved in the regulation of downstream PI3K/AKT signaling and cellular responses such as apoptosis [51]. TLR-mediated signaling pathways play important roles in the immune response during mastitis, while NF-κB is a key regulator of inflammation-associated cytokine production [52,53]. In the present study, *S. agalactiae* infection increased the expression of TLR2, PI3K, and NF-κB and enhanced AKT phosphorylation in mammary tissues. RA treatment partially reversed these infection-associated changes, as evidenced by reduced TLR2, PI3K, and NF-κB expression and decreased AKT phosphorylation. AKT is an important signaling molecule involved in regulating inflammation and can interact with NF-κB signaling [54,55]. The present findings suggest that *S. agalactiae* infection activates the PI3K/AKT and NF-κB signaling pathways in mammary tissues, whereas RA treatment attenuates this activation. This effect may contribute to the reduction in inflammatory responses observed in the RA-treated groups. Consistent with previous studies, RA has been reported to suppress LPS-induced AKT activation and NF-κB nuclear translocation in human umbilical vein endothelial cells [56]. In addition, RA derivatives have been reported to modulate the AKT/NF-κB pathway in tumor cells, thereby attenuating inflammation and cellular damage [56,57]. Together with findings from the mouse mastitis model, these results suggest that modulation of the PI3K/AKT and NF-κB signaling pathways may contribute to the protective effects of RA against *S. agalactiae*-induced mastitis.

In addition to its effects on inflammatory signaling, the present study showed that RA attenuated abnormal apoptosis-related changes in mammary tissues following *S. agalactiae* infection, suggesting a potential role in maintaining mammary tissue homeostasis. In our previous study, transcriptomic analysis showed that *S. agalactiae* infection of mammary epithelial cells resulted in significant enrichment of differentially expressed genes in cancer-related pathways, including the p53 signaling pathway, with Bim (BCL2L11) and RUNX1 identified as potential candidate genes [13]. Previous studies have also demonstrated that Bim participates in the apoptosis of mammary epithelial cells during mammary gland involution [58]. In the present study, the mRNA expression levels of RUNX1 and Bim were significantly increased in the mammary glands of postpartum/lactating mice infected with *S. agalactiae*, whereas their protein expression levels were decreased. In addition, the phosphorylation level of Bim was significantly increased in the model group and was reduced following RA treatment. These findings indicate that *S. agalactiae* infection alters the transcriptional, translational, and post-translational regulation of apoptosis-related proteins, whereas RA partially reverses these changes. Previous studies have reported that Bim expression can be regulated by the PI3K/AKT signaling pathway [59]. Consistent with this relationship, the present study showed that *S. agalactiae* infection was accompanied by activation of PI3K/AKT signaling and reduced Bim protein expression, whereas RA treatment partially reversed these changes. Furthermore, the decrease in total p53 protein expression and the increase in p53 phosphorylation observed following RA treatment suggest that RA may modulate p53-related apoptotic signaling in mammary tissues. Taken together, these findings suggest that the protective effects of RA against *S. agalactiae*-induced mastitis may involve coordinated modulation of inflammatory and apoptosis-related signaling pathways, including the TLR2/NF-κB, PI3K/AKT, and p53/Bim-associated pathways.

In addition, the present study primarily focused on the anti-inflammatory effects of RA in *S. agalactiae*-induced mouse mastitis, whereas a comprehensive evaluation of its systemic safety profile was not performed. Although RA treatment alleviated mammary tissue injury and inflammatory responses under the experimental conditions, its potential effects on other organs, particularly the liver and kidney, remain to be further investigated. Given that different doses of RA were administered in the present study, future studies should include systematic toxicity assessments, such as serum biochemical analyses and histological evaluation of major organs, to further define the safety profile and optimal application range of RA. Another limitation of the present study is the absence of pharmacokinetic and mammary-gland tissue-distribution data. Therefore, we cannot definitively confirm whether intraperitoneally administered RA (25, 50, 100 mg/kg) achieves local mammary-tissue concentrations equivalent to its in vitro MIC of 8 mg/mL against *S. agalactiae*. The in vivo protective effect of RA may arise predominantly from host-modulating anti-inflammatory and anti-virulence activities rather than direct local bacterial-growth inhibition. Further LC-MS-based tissue-distribution studies are warranted in future work. These investigations will provide important information for the future development of RA as a potential plant-derived antimicrobial and anti-inflammatory agent for mastitis management.

## 5. Conclusions

In conclusion, RA exhibited promising antibacterial and anti-inflammatory activities against *S. agalactiae*-induced mastitis. At the MIC, RA inhibited the growth of *S. agalactiae* and impaired key bacterial virulence-associated phenotypes, including adhesion, capsular polysaccharide production, and biofilm formation. In the mouse model of *S. agalactiae*-induced mastitis, RA treatment alleviated mammary tissue damage, reduced inflammatory cell infiltration and the production of pro-inflammatory cytokines, and restored IL-10 expression. These protective effects were accompanied by attenuation of TLR2/NF-κB and PI3K/AKT signaling and modulation of apoptosis-related factors, including Bim and p53. Together, these findings suggest that RA may exert its protective effects against *S. agalactiae*-induced mastitis through coordinated regulation of bacterial virulence and host inflammatory and apoptosis-related responses. Although further studies are required to elucidate the direct molecular targets of RA and to validate its efficacy and safety in relevant livestock models, the present findings provide a basis for further evaluating RA as a potential plant-derived antimicrobial and anti-inflammatory agent for the prevention and management of mastitis.

## Figures and Tables

**Table 1:** The number of somatic cells in milk.

Control	Model	25 mg/kg	50 mg/kg	100 mg/kg	*p*-Value
3.67 ± 0.49 ^c^	17.33 ± 1.43 ^a^	8.33 ± 0.43 ^bd^	11.5 ± 0.62 ^b^	5.67 ± 0.42 ^cd^	<0.001

Note: Different letters indicate *p* < 0.05. The unit of measurement is cells/10 μL. 6 independent biological replicates were performed, with 3 technical replicates per biological replicate; One-way ANOVA followed by Tukey’s HSD test.

**Table 2:** The number of somatic cells in the blood.

Cell Type	Control	Model	25 mg/kg	50 mg/kg	100 mg/kg	*p*-Value
Neutrophils	0.02 ± 0.01 ^b^	0.16 ± 0.12 ^a^	0.15 ± 0.11 ^a^	0.02 ± 0.01 ^b^	0.03 ± 0.03 ^b^	0.002
Lymphocytes	3.05 ± 0.77 ^d^	10.22 ± 0.43 ^a^	5.81 ± 0.76 ^b^	4.79 ± 0.74 ^c^	3.28 ± 0.63 ^d^	<0.001
Intermediate cells	0.06 ± 0.02 ^c^	0.27 ± 0.05 ^a^	0.13 ± 0.06 ^b^	0.06 ± 0.02 ^c^	0.06 ± 0.02 ^c^	<0.001
Leukocytes	3.12 ± 3.12 ^d^	10.64 ± 0.57 ^a^	6.08 ± 0.60 ^b^	4.87 ± 0.75 ^c^	3.33 ± 0.62 ^d^	<0.001

Note: Different letters in the same row indicate *p* < 0.05. The values are presented as the mean and SD. The units of measurement are cells × 10^9^/L. 6 independent biological replicates were performed, with 3 technical replicates per biological replicate; One-way ANOVA followed by Tukey’s HSD test.

## Data Availability

All data generated or analyzed during this study are included in this published article.

## Supplementary Materials

The following supporting information can be downloaded at: https://www.mdpi.com/article/10.3390/ani16182917/s1, Table S1: Product Information of ELISA Kits; Table S2: Primer information; Table S3: Product Specifications of Antibodies.
