# Supplementary material for: Antibacterial Activity of Rosmarinic Acid Against Streptococcus agalactiae and Its Anti-Inflammatory Effects in Mouse Mastitis
Source: Animals (Basel). 2026 Sep 16;16(18):2917. doi: 10.3390/ani16182917 (PMC13603107; doi:10.3390/ani16182917)
Supplement: Supplementary file 1 [file animals-16-02917-s001.zip › Supplement Table S3.pdf]

Table S3 Product Specifications of Antibodies

Antibody	Company	Product Number
TNF-α	Affinity Biosciences LTD	AF7014
P53		AF0879
p-P53		AF3073
TLR2		DF7002
PI3K		AF6241
Bim		DF6093
p-Bim		AF8248
RUNX1		AF6379
p-RUNX1		AF3378
NF-κB	Proteintech Group, Inc.	10745-1-AP
AKT		60203-2-Ig
p-AKT		66444-1-Ig
HRP-Goat anti Rabbit		SA00001-2
HRP-Goat anti Mouse		SA00001-1
GAPDH	Hangzhou HuaAn Biotechnology Co., Ltd.	ET1601-4
