# Supplementary material for: Antibacterial Activity of Rosmarinic Acid Against Streptococcus agalactiae and Its Anti-Inflammatory Effects in Mouse Mastitis
Source: Animals (Basel). 2026 Sep 16;16(18):2917. doi: 10.3390/ani16182917 (PMC13603107; doi:10.3390/ani16182917)
Supplement: Supplementary file 1 [file animals-16-02917-s001.zip › Supplement Table S2.pdf]

Table S2 Primer information

Gene	GeneBank NO.	Primer sequence	Product Size (bp)
16S	NZ_CP012480.1	GCTCACCAAGGCGACGATACATAG<br>GCGTTGCTCGGTCAGACTTCC	135
CAMP	NZ_CP012480.1	AAGCCCAGCAAATGGCTCAA<br>CTGTCTCAGAGTTGGCACGC	156
BIBA	NZ_CP012480.1	AGCTAAGCAGTCAAGACGTTCTC<br>TCAGTTGCGTGGCTTGTGTT	146
FbsA	NZ_CP012480.1	AGCGTCGTCAACGTGATGCG<br>ACGCTCTAGAACATTGCCTTGGC	149
FbsB	NZ_CP012480.1	ACTGCGCAAACTTCTGTCCA<br>GCGAGGTCATTTCCGCAGTT	166
PI-1	NZ_CP012480.1	AGTGCTGATGCAACACCTGA<br>TGTACGACGCTTCATCCCCT	141
PI-2a	NZ_CP012480.1	GCGGCTACACTCCAGAATACG<br>CAGTTGCTGCACCTGCTTTAC	228
CylE	NZ_CP012480.1	AAGTCGTAGTGGACAGGCAATCAC<br>GGAGGTGACTACGCAATGATCGAC	200
CpsA	NZ_CP012480.1	ACAACGCTTCACTGTCGAGTCAC<br>AGCTCCTGGCATTGCATATGAAGG	82
HylB	NZ_CP012480.1	GCTTCAACCGCAACTGCAACAG<br>CGCTTGTATGTGACCAGCTTCCAG	139
Sip	NZ_CP012480.1	GACACCAGAAGCAGCAACAA<br>GTTTCAGCAGCAACAGAAGC	240
CPSE	NZ_CP012480.1	CAGAAGCGACGCCTTAGTTT<br>GACCACCACTTGAACCAACC	210
SCPB	NZ_CP012480.1	TCAGAGTCTATCTCGGGCAAGT<br>TCACGCCACGAATATCATCAA	95
GAPDH	NM_001411845.1	GCCCAGAACATCATCCCTGCAT<br>GCCTGCTTCACCACCTTCTTGA	188
IL-1α	NM_010554.4	GAGAGCCGGGTGACAGTATC<br>ATGGGTTATGGACTGCAGGT	111
IL-1β	NM_008361.4	GATGAAGGGCTGCTTCCAAACCT<br>GAAAGACACAGGTAGCTGCCACA	151
IL-6	NM_001314054.1	GGATACCACTCCCAACAGACCTG<br>TGTTCTTCATGTACTCCAGGTAGCT	278
TNF-α	NM_013693.3	GGTGCCTATGTCTCAGCCTCTTC<br>TGATCTGAGTGTGAGGGTCTGGG	161
NF-κB	NM_001402548.1	GATACCACCAAGACACACCCCAC<br>CTCCAGGTCTCGCTTCTTCACAC	225
p53	XM_030245922.1	AGAGACCGCCGTACAGAAGAAGA<br>GACTGGCCCTTCTTGGTCTTCAG	290
Bim	XM_006498614.4	GCCCCTACCTCCCTACAGAC	129

		TGGGGATCTGGTAGCAAAAG	
TLR2	NM_011905.3	TTGCTCCTGCGAACTCCTAT<br>GCTTTCTTGGGCTTCCTCTT	294
PI3K	NM_001077495.2	AGGAGGAGGTTGGAAGAAGAC<br>GCGTCAGCCACATCAAGTATT	133
Akt	NM_001331107.2	CCACGCTACTTCCTCCTCAA<br>CGTCTCTTCTTCCTGCCTCT	282
RNUX1	NM_001111021.2	TTCATGAGAGATGCCAGCAC<br>GTAGCACGGAGCAGAGGAAG	220
