# Supplementary material for: Antibacterial Activity of Rosmarinic Acid Against Streptococcus agalactiae and Its Anti-Inflammatory Effects in Mouse Mastitis
Source: Animals (Basel). 2026 Sep 16;16(18):2917. doi: 10.3390/ani16182917 (PMC13603107; doi:10.3390/ani16182917)
Supplement: Supplementary file 1 [file animals-16-02917-s001.zip › Supplement Table S1.pdf]

Table S1 Product Information of ELISA Kits

Elisa Kit	Company	Product Number
IL-1α	Jiangsu Enzyme-linked Industrial Co., Ltd.	MM-0168M2
TNF-a		MM-0040M2
IL-1β		MM-0040M2
IL-6		MM-0163M2
IL-10		MM-0176M2
